# Supplementary material for: KLF11 deficiency enhances chemokine generation and fibrosis in murine unilateral ureteral obstruction
Source: PLoS One. 2022 Apr 12;17(4):e0266454. doi: 10.1371/journal.pone.0266454 (PMC9004740; doi:10.1371/journal.pone.0266454)
Supplement: S1 Table — (PDF) [file pone.0266454.s001.pdf]

S1 Table. Differentially expressed genes of TGF-β/BMP/Fibrotic pathways between the KLF11 KO vs WT.

	WT-Sham	KLF11 KO-Sham	WT-UUO	KLF11 KO-UUO	
**BMP Family (Bone Morphogenetic Proteins)**					
Bambi	1±0.13	1.1±0.16 (a)ns	1.7±0.16 (b)ns	**3.1±0.79 (c)***	(d)ns
Bmp2	1±0.15	0.89±0.2 (a)ns	**2.5±0.34 (b)***	**2.8±0.44 (c)****	(d)ns
Bmp6	1±0.13	0.87±0.13 (a)ns	1.2±0.13 (b)ns	**1.5±0.18 (c)***	(d)ns
Bmper	1±0.28	3.9±1.8 (a)ns	18±8.1 (b)ns	18±4.6 (c)ns	(d)ns
Bmp4	1±0.067	1.4±0.22 (a)ns	0.96±0.067 (b)ns	1.4±0.24 (c)ns	(d)ns
**Cell Adhesion Molecules**					
Itgb1	1±0.03	1.1±0.063 (a)ns	**3.1±0.25 (b)******	**3.4±0.096 (c)******	(d)ns
Itga2	1±0.089	1.6±0.74 (a)ns	**13±2.9 (b)*****	**16±1.6 (c)*****	(d)ns
Itgb3	1±0.091	1.2±0.49 (a)ns	**6.5±1 (b)******	**6.5±0.8 (c)*****	(d)ns
Itgb5	1±0.11	1.1±0.1 (a)ns	**2.7±0.24 (b)*****	**3.6±0.4 (c)******	(d)ns
Itgb6	1±0.15	0.84±0.091 (a)ns	**1.5±0.097 (b)****	**1.7±0.098 (c)******	(d)ns
Itgb8	1±0.097	1.1±0.08 (a)ns	0.73±0.068 (b)ns	0.88±0.074 (c)ns	(d)ns
**Cell cycle progression**					
Cdkn1a	1±0.15	0.74±0.32 (a)ns	**7.3±0.64 (b)******	**6.5±0.89 (c)******	(d)ns
Gadd45b	1±0.23	1.4±0.24 (a)ns	**6.2±0.53 (b)******	**5.7±0.58 (c)******	(d)ns
Cdkn2b	1±0.24	1.2±0.26 (a)ns	6.3±0.73 (b)ns	**17±5.2 (c)****	(d)ns
**ECM Remodeling Enzymes**					
Lox	1±0.19	0.9±0.36 (a)ns	**20±4.1 (b)*****	**25±3.1 (c)******	(d)ns
Mmp1a	1±0.17	**1.7±0.19 (a)***	**0.38±0.031 (b)****	**0.38±0.048 (c)******	(d)ns
Mmp2	1±0.1	1.5±0.29 (a)ns	**21±5.2 (b)****	**32±3.8 (c)******	(d)ns
Mmp3	1±0.19	0.68±0.19 (a)ns	**40±7.2 (b)******	**29±4.3 (c)****	(d)ns
Mmp14	1±0.13	0.77±0.15 (a)ns	**24±5 (b)*****	**26±3.9 (c)*****	(d)ns
Plat	1±0.054	1.1±0.18 (a)ns	**4.2±0.38 (b)******	**4.2±0.32 (c)******	(d)ns
Serpine1	1±0.095	1.2±0.2 (a)ns	**31±5.3 (b)******	**29±2.4 (c)******	(d)ns
Serpinh1	1±0.07	0.99±0.11 (a)ns	**6.2±0.84 (b)******	**7.1±0.66 (c)******	(d)ns
Timp1	1±0.21	3±2.6 (a)ns	**284±73 (b)*****	**199±28 (c)***	(d)ns
Timp2	1±0.075	0.89±0.13 (a)ns	**5.2±0.72 (b)******	**6.3±0.53 (c)******	(d)ns
Mmp8	1±0.38	1.5±0.99 (a)ns	17±7.4 (b)ns	6.4±1.2 (c)ns	(d)ns
Mmp9	1±0.13	1.2±0.54 (a)ns	13±5.1 (b)ns	14±4.8 (c)ns	(d)ns
Mmp13	1±0.11	0.91±0.19 (a)ns	**2.4±0.49 (b)***	1.4±0.33 (c)ns	(d)ns
**Growth Factors**					
Edn1	1±0.088	0.9±0.12 (a)ns	**32±4.8 (b)******	**38±2.9 (c)******	(d)ns
Egf	1±0.1	1.1±0.1 (a)ns	**0.096±0.063 (b)******	**0.044±0.007 (c)******	(d)ns
Tdgf1	1±0.068	1±0.091 (a)ns	**7.6±1 (b)******	**7.2±0.42 (c)******	(d)ns
Vegfa	1±0.06	1.2±0.074 (a)ns	**0.77±0.039 (b)***	**0.82±0.043 (c)*****	(d)ns
Agt	1±0.099	0.94±0.11 (a)ns	1.4±0.14 (b)ns	1.1±0.037 (c)ns	(d)ns
Gdf1	1±0.27	7.8±3.3 (a)ns	3.3±0.68 (b)ns	5.2±0.78 (c)ns	(d)ns
Gdf5	1±0.27	1.3±0.27 (a)ns	1.9±0.41 (b)ns	2.3±0.54 (c)ns	(d)ns
Gdf7	1±0.27	**2.5±0.5 (a)***	1.8±0.4 (b)ns	2±0.59 (c)ns	(d)ns
Igfbp3	1±0.12	1±0.1 (a)ns	0.91±0.073 (b)ns	2.2±1 (c)ns	(d)ns
**Pro-Fibrotic**					
Acta2	1±0.083	**0.54±0.048 (a)*****	**5.4±0.81 (b)******	**4.9±0.58 (c)******	(d)ns
Ctgf	1±0.08	0.78±0.13 (a)ns	**3.8±0.61 (b)****	**3.9±0.67 (c)*****	(d)ns
**SMAD Family**					
SMAD7	1±0.052	**1.4±0.18 (a)***	**2±0.13 (b)****	**2.6±0.32 (c)****	(d)ns
SMAD6	1±0.13	1.4±0.34 (a)ns	1.4±0.14 (b)ns	2.1±0.32 (c)ns	(d)ns

**TGF-β Superfamily Members**					
Amhr2	1±0.35	3.7±1.4 (a)ns	0.73±0.2 (b)ns	**1±0.21 (c)***	(d)ns
Cav1	1±0.11	1.1±0.12 (a)ns	**1.5±0.14 (b)***	**1.9±0.12 (c)*****	(d)ns
Chrd	1±0.058	**1.7±0.2 (a)****	**2.4±0.33 (b)****	**3.2±0.25 (c)****	(d)ns
Dcn	1±0.079	0.93±0.18 (a)ns	5±0.8 (b)ns	**7.7±1.8 (c)*****	(d)ns
Eng	1±0.097	0.92±0.12 (a)ns	**2.2±0.13 (b)*****	**2.6±0.27 (c)******	(d)ns
Grem1	1±0.18	0.74±0.22 (a)ns	**38±8.3 (b)***	**49±11 (c)****	(d)ns
TGF-β1i1	1±0.086	0.9±0.15 (a)ns	**5.2±0.53 (b)******	**5.9±0.59 (c)******	(d)ns
Tgif1	1±0.06	1±0.079 (a)ns	**7.6±0.9 (b)******	**7.2±0.37 (c)******	(d)ns
Thbs1	1±0.082	1±0.12 (a)ns	**7.1±0.76 (b)******	**8.2±0.62 (c)******	(d)ns
Thbs2	1±0.051	0.75±0.14 (a)ns	**31±8 (b)****	**32±6 (c)****	(d)ns
Inha	1±0.22	**4.8±1.4 (a)***	**5.3±0.88 (b)***	7.8±1.1 (c)ns	(d)ns
Inhba	1±0.22	9.2±7.5 (a)ns	**56±13 (b)*****	**47±6.4 (c)***	(d)ns
Inhbe	1±0.3	2±0.81 (a)ns	0.48±0.15 (b)ns	1.2±0.48 (c)ns	(d)ns
Nog	1±0.15	8.4±4.4 (a)ns	1±0.31 (b)ns	1.4±0.34 (c)ns	(d)ns
TGF-βrap1	1±0.074	1.4±0.26 (a)ns	0.97±0.1 (b)ns	1.3±0.16 (c)ns	(d)ns
TGF-βr3	1±0.1	1.4±0.36 (a)ns	1.5±0.17 (b)ns	2±0.38 (c)ns	(d)ns
**TNF receptor superfamily**					
Tnfsf14	1±0.21	0.79±0.16 (a)ns	4.1±0.87 (b)ns	**7.5±2 (c)****	(d)ns
**Transcription Factors**					
Atf4	1±0.061	**1.4±0.15 (a)***	2±0.1 (b)ns	**3.5±0.95 (c)***	(d)ns
Cebpb	1±0.071	0.83±0.17 (a)ns	**2.5±0.23 (b)******	**2.5±0.16 (c)******	(d)ns
Fos	1±0.2	1.5±0.5 (a)ns	**17±2.1 (b)******	**14±1.8 (c)******	(d)ns
Ifrd1	1±0.028	1.3±0.17 (a)ns	3.6±0.46 (b)ns	**5.5±1.7 (c)***	(d)ns
Jun	1±0.058	1.1±0.13 (a)ns	**5.6±0.65 (b)******	**6.2±0.62 (c)******	(d)ns
Myc	1±0.078	0.97±0.15 (a)ns	**7.5±0.67 (b)******	**8.5±0.91 (c)******	(d)ns
Snai1	1±0.065	1.1±0.26 (a)ns	**6.9±0.98 (b)******	**7.5±1 (c)******	(d)ns
Sox4	1±0.13	1.6±0.32 (a)ns	**13±1.8 (b)****	**16±3.2 (c)*****	(d)ns
Id1	1±0.15	1.1±0.29 (a)ns	1.1±0.063 (b)ns	1±0.16 (c)ns	(d)ns
Id2	1±0.12	**1.6±0.17 (a)***	1±0.073 (b)ns	1.6±0.34 (c)ns	(d)ns
Mecom	1±0.1	1.5±0.26 (a)ns	1.5±0.16 (b)ns	2±0.2 (c)ns	(d)ns
**ECM Structural Constituents**					
Col1a1	1±0.11	1±0.21 (a)ns	**35±6.1 (b)*****	**40±7.3 (c)******	(d)ns
Col3a1	1±0.11	0.68±0.16 (a)ns	**35±5.3 (b)******	**37±6.1 (c)******	(d)ns
**Other Genes**					
Bcl2	1±0.078	**1.6±0.29 (a)***	**2.2±0.27 (b)****	**2.6±0.13 (c)***	(d)ns
Herpud1	1±0.091	1.1±0.15 (a)ns	**0.39±0.069 (b)*****	**0.35±0.03 (c)******	(d)ns
Bglap2	1±0.16	1.3±0.34 (a)ns	0.76±0.097 (b)ns	1.6±0.51 (c)ns	(d)ns
Fst	1±0.26	**2±0.3 (a)***	2±0.24 (b)ns	4.1±1 (c)ns	(d)ns

S1Table: Differentially expressed genes TGF-β/BMP pathway and fibrotic response in Sham mice compared to UUO mice. Gene expression analysis was performed employing the pathway Detect RNA array. The table showed the differentially expressed genes by RTPCR after 9 days of Surgery Sham/UUO. Statistical significance was determined by Student's t-test. **(a)** KLF11 KO-Sham compared with WT-Sham, **(b)** WT-UUO compared with WT-Sham, **(c)** KLF11 KO-UUO compared with KLF11 KO-Sham, **(d)** KLF11 KO-UUO compared with WT-UUO. Values are means

± SEM. p values ≤0.05 were considered as significant (GraphPad Software, La Jolla, CA). Statistically significant values are highlighted in bold: *$p \leq 0.05$; **$p \leq 0.01$; *** $p \leq 0.001$; ****$p \leq 0.0001$
