# Supplementary material for: KLF11 deficiency enhances chemokine generation and fibrosis in murine unilateral ureteral obstruction
Source: PLoS One. 2022 Apr 12;17(4):e0266454. doi: 10.1371/journal.pone.0266454 (PMC9004740; doi:10.1371/journal.pone.0266454)
Supplement: S3 Table — (PDF) [file pone.0266454.s003.pdf]

S3 Table. Renal function parameters in KLF11 KO and WT mice

	WT-Sham	KLF11 KO-Sham	WT-UUO	KLF11 KO-UUO	
**ALB**	3.9± 0.1	3.6±0.1 (a)ns	3.7±0.1 (b)ns	3.3 ±0.2 (c)ns	(d)ns
**BUN**	20.4± 1.3	15.2±4.7 (a)ns	33.0±2.5 (b)*	22.67±3.3 (c)ns	(d)ns
**CREA**	<0.1	<0.1 (a)ns	<0.1 (b)ns	0.114 (c)ns	(d)ns
**GLU**	182.4± 23.0	174.2±23.1 (a)ns	189.86±20.9 (b)ns	166.67±22.3 (c)ns	(d)ns

S3 Table: Renal function in KLF11KO and WT mice. The table showed Albumin (ALB), Blood Urea Nitrogen (BUN), Creatinine (CREA) and Glucose (GLU) after 9 days of Surgery Sham/UUO. Statistical significance was determined by Student's t-test. (a) KLF11 KO-Sham (n=4) compared with WT-Sham, (b) WT-UUO (n=7) compared with WT-Sham (n=4), (c) KLF11 KO-UUO (n=6) compared with KLF11 KO-Sham, (d) KLF11 KO-UUO compared with WT-UUO. Values are means ± SEM. p values ≤0.05 were considered as significant. Statistically significant values are highlighted in bold: *p ≤ 0.05; **p ≤ 0.01; *** p ≤ 0.001; ****p ≤ 0.0001, ns: not significant.
