# Supplementary material for: KLF11 deficiency enhances chemokine generation and fibrosis in murine unilateral ureteral obstruction
Source: PLoS One. 2022 Apr 12;17(4):e0266454. doi: 10.1371/journal.pone.0266454 (PMC9004740; doi:10.1371/journal.pone.0266454)
Supplement: S4 Table — (PDF) [file pone.0266454.s004.pdf]

S4 Table. CD3 and CD163 markers quantification in KLF11 KO and WT

	WT-Sham	KLF11 KO-Sham	WT-UUO	KLF11 KO-UUO	
**CD163**	0.034± 0.012	0.015± 0.003 (a)ns	0.136± 0.025 (b)**	0.081± 0.014 (c)***	(d)ns
**CD3**	0.763± 0.076	0.623± 0.093 (a)ns	4.130± 0.568 (b)**	3.340± 0.319 (c)***	(d)ns

S4 Table: CD3 and CD163 quantification in KLF11 KO and WT. The table showed the quantitative analysis of the percent cortical surface area staining positively for CD3 and CD163 after 9 days of Surgery Sham/UUO. Statistical significance was determined by Student's t-test. (a) KLF11 KO-Sham compared with WT-Sham, (b) WT-UUO compared with WT-Sham, (c) KLF11 KO-UUO compared with KLF11 KO-Sham, (d) KLF11 KO-UUO compared with WT-UUO. Values are means ± SEM. p values ≤0.05 were considered as significant. Statistically significant values are highlighted in bold: *p ≤ 0.05; **p ≤ 0.01; *** p ≤ 0.001; ****p ≤ 0.0001, ns: not significant.
