# Supplementary material for: KLF11 deficiency enhances chemokine generation and fibrosis in murine unilateral ureteral obstruction
Source: PLoS One. 2022 Apr 12;17(4):e0266454. doi: 10.1371/journal.pone.0266454 (PMC9004740; doi:10.1371/journal.pone.0266454)
Supplement: Attachment — Submitted filename: PONE Response to Reviewers.pdf [file pone.0266454.s005.pdf]

PONE-D-21-23487
KLF11 deficiency enhances chemokine generation and activates the TGF-β/BMP fibrotic pathway in murine unilateral ureteric obstruction
PLOS ONE

We would like to thank the reviewers for their thoughtful comments provided in review of our manuscript, “KLF11 deficiency enhances chemokine generation and activates the TGF-β/BMP fibrotic pathway in murine unilateral ureteric obstruction”. We have conducted additional experiments and have made a number of clarifications in response to the constructive comments provided. A summary of the changes is provided below:

Funding information should not be included in the Acknowledgements Section of the Manuscript.

We have removed the statement indicating that the Department of Laboratory Medicine and Pathology provided institutional support for these studies in the acknowledgements section. As suggested, we wish to update the Funding Statement to indicate:

"This work was funded by the Department of Laboratory Medicine and Pathology, Mayo Clinic College of Medicine. The funders had no role in study design, data collection and analysis, decision to publish, or preparation of the manuscript."

We note that you have included the phrase “data not shown” in your manuscript. Unfortunately, this does not meet our data sharing requirements. PLOS does not permit references to inaccessible data. We require that authors provide all relevant data within the paper, Supporting Information files, or in an acceptable, public repository. Please add a citation to support this phrase or upload the data that corresponds with these findings to a stable repository (such as Figshare or Dryad) and provide and URLs, DOIs, or accession numbers that may be used to access these data. Or, if the data are not a core part of the research being presented in your study, we ask that you remove the phrase that refers to these data

We have provided all data in supporting information files.

Please include your full ethics statement in the ‘Methods’ section of your manuscript file. In your statement, please include the full name of the IRB or ethics committee who approved or waived your study, as well as whether or not you obtained informed written or verbal consent. If consent was waived for your study, please include this information in your statement as well

This study does not involve human subjects. We have included the statement that “all animal procedures were approved by the Mayo Clinic Institutional Animal Care and Use Committee (IACUC) prior to conducting any experiments. These animal procedures were conducted in accordance with then National Institutes of Health Guide for the Care and Use of Laboratory Animals”.

Dear authors,

would you please provide renal function parameters (Crea, BUN) and Masson Trichrome staining to judge injury?

In the unilateral ureteral obstruction model, the contralateral kidney provides normal renal function. Therefore, changes in serum creatinine are not anticipated. We have done additional studies to measure BUN and albuminuria and have provided this information in a supplementary table. We have used Sirius Red staining to provide a quantitative assessment of extracellular matrix deposition. Sirius Red staining provides a more accurate assessment of matrix deposition than trichrome staining, as edema and non-collagenous components can stain blue with a trichrome stain. We have compared Sirius Red staining with trichrome staining in our previously published study [1].

Reviewer #1: In this study, the authors describe the pathological roles of KLF11 in renal fibrosis. In particular, KLF11 KO mice showed increased kidney injury and fibrosis, accompanied by upregulation of expression of pro-fibrotic and pro-inflammatory genes. There are serious concerns to be addressed.

1. The quality of H&E staining, immunohistochemical staining and heatmaps is of poor quality. For example, I can't identify the specific marker positive cells in histological images and the gene name on the heatmaps.

We have revised the figures to provide higher resolution images of the immunohistochemical staining and have enlarged the annotations to the heatmaps.

2. The authors need to indicate the expression of other KLF family factors in KLF11 KO mice with or without UUO, because deletion of KLF11 may compensatory change the expression of other KLF family factors.

We performed additional studies employing RNASeq to assess other KLF family members in KLF11 KO mice with or without UUO. Our data are summarized in a heatmap showing relative expression of the KLF family members in sham and UUO mice (Fig 1 B). We see largest induction of KLF14 and KLF 16 in KLF11 KO UUO compared to WT UUO; other KLF members showed modest changes according to genotype.

3. Although the authors demonstrated that macrophage infiltration and fibrosis was suppressed in the kidneys of KLF11 global KO mice, it is unclear which KLF11-expressing cells contribute to these phenotypes. Which cells are expressing KLF11 in murine normal kidneys and injured kidneys?

We performed additional immunohistochemical studies to identify KLF11 staining cells. We found nuclear staining for KLF11, primarily in proximal and distal tubular epithelial cells, with focal glomerular staining of visceral and parietal epithelial cells. We observed stronger staining in WT mice subjected to UUO, compared to sham, consistent with our RNASeq data showing induction of KLF11 in WT UUO mice compared to sham.

4. It would be interesting to indicate the association of KLF11 with kidney diseases. For example, how does endogenous KLF11 mRNA and protein expression change after UUO?

Although other KLF family members have been implicated in human and experimental kidney disease, to the best of our knowledge, this is the first report associating KLF11 with kidney disease. At the RNA and protein level, as assessed by RNASeq and immunohistochemistry, respectively, we do demonstrate that, in WT mice, KLF11 is induced with UUO.

5. The author describe that the expression of CD3 or CD163-positive cells was examined in methods and results section and the expression of CD68-positive cells was examined in methods section. However, I can't find these data in this manuscript.

We have provided these data in a supplemental table.

Reviewer #2: 1. In Results Genetic inactivation of KLF11 increases renal injury in UUO model, authors only can get conclusion that genetic inactivation of KLF11 contribute to tubular atrophy, because tubular atrophy can not be equal to renal injury. I suggest authors detect some renal injury biomarkers in this part.

Tubular atrophy is a well-recognized feature of chronic renal injury. For example, ct scores are an integral component of chronic tubular injury scoring according to the Banff Classification of transplant pathology. [2]. We have extensively employed tubular atrophy as an index of chronic tubular injury in our previous publications involving both human and experimental studies [3] [4] [5] [6]. As expected with a unilateral injury model, we did not detect significant differences in serum creatinine among the experimental groups.

2. The title that KLF11 deficiency enhances chemokine generation and activates the TGF-β/BMP fibrotic pathway in murine unilateral ureteric obstruction is not appropriate, because KLF11 deficiency enhances chemokine generation, and increase inflammation and pro-inflammation cytokines production to result in renal damage from the text, but can not attribute to activate the TGF-β/BMP fibrotic pathway specifically.

KLF11 was originally described as a Transforming Growth Factor Beta inducible immediate early gene (TIEG) [7]. Based on this consideration, it was reasonable to focus on the TGF-beta/SMAD pathway; we have shown significant perturbations in this pathway in KLF11 deficient mice. Nevertheless, we have removed "activation of the TGF-beta/BMP fibrotic pathway" from the title.

3. From the whole study, authors just observed a phenomenon that KLF11 deficiency results in renal

injury, accompanied with chemokine generation enhanced and inflammation and pro-inflammation cytokines production increased, but no specific mechanism.

To our knowledge, this is the first report linking KLF11 deficiency to renal injury. Characterization of differentially regulated pathways is an important first step in the development of a mechanistic hypothesis whereby KLF11 deficiency results in renal injury. Based on initial characterization of KLF11 as a TIEG, we hypothesized that the renal damage was associated with alterations in TGF-beta signaling. Futures studies will focus on a mechanism whereby KLF11 deficiency promotes kidney injury, with a focus on the TGF-beta pathway.

---

1. Diaz Encarnacion M, Griffin M, Slezak J, Bergstralh E, Stegall M, Velosa J, et al. Correlation of quantitative digital image analysis with glomerular filtration rate in CAN. Am J Transplantation. 2004;4(2):248-56. PubMed PMID: 14974947.
2. Racusen LC, Solez K, Colvin RB, Bonsib SM, Castro MC, Cavallo T, et al. The Banff 97 working classification of renal allograft pathology. Kidney International. 1999;55(2):713-23.
3. Helgeson ES, Mannon R, Grande J, Gaston RS, Cecka MJ, Kasiske BL, et al. i-IFTA and chronic active T cell-mediated rejection: A tale of 2 (DeKAF) cohorts. Am J Transplant. 2021;21(5):1866-77. Epub 2020/10/15. doi: 10.1111/ajt.16352. PubMed PMID: 33052625.
4. Grande JP, Helgeson ES, Matas AJ. Correlation of Glomerular Size With Donor-Recipient Factors and With Response to Injury. Transplantation. 2021;105(11):2451-60. Epub 2020/12/05. doi: 10.1097/TP.0000000000003570. PubMed PMID: 33273317; PubMed Central PMCID: PMCPMC8166916.
5. Kashyap S, Osman M, Ferguson CM, Nath MC, Roy B, Lien KR, et al. Ccl2 deficiency protects against chronic renal injury in murine renovascular hypertension. Scientific Reports. 2018;8(1):8598.
6. Kashyap S, Boyilla R, Zaia PJ, Ghossan R, Nath KA, Textor SC, et al. Development of renal atrophy in murine 2 kidney 1 clip hypertension is strain independent. Res Vet Sci. 2016;107:171-7.
7. Lin L, Mahner S, Jeschke U, Hester A. The Distinct Roles of Transcriptional Factor KLF11 in Normal Cell Growth Regulation and Cancer as a Mediator of TGF-beta Signaling Pathway. Int J Mol Sci. 2020;21(8). Epub 2020/04/26. doi: 10.3390/ijms21082928. PubMed PMID: 32331236; PubMed Central PMCID: PMCPMC7215894.
