# Supplementary material for: KLF11 deficiency enhances chemokine generation and fibrosis in murine unilateral ureteral obstruction
Source: PLoS One. 2022 Apr 12;17(4):e0266454. doi: 10.1371/journal.pone.0266454 (PMC9004740; doi:10.1371/journal.pone.0266454)
Supplement: Attachment — Submitted filename: Summary of changes to manuscript.docx [file pone.0266454.s006.docx]

*Reviewer #1: In this revision, the authors have satisfactorily addressed my previous comments, and I do not have any additional comments.*

We wish to thank this reviewer for the constructive comments that have improved the manuscript.

Reviewer #3:

*1. (Figure 1) Expression of KLF11 by the specific cells described in lines 177-178 of the manuscript (tubular epithelial cells, podocytes, and infiltrating mononuclear cells) is difficult to discern in the light micrograph of the WT-UUO kidney section. Is the KLF11 shown in brown? Are glomeruli shown in these micrographs? I would recommend that the authors either provide additional micrographs that more clearly portray the expression and/or use arrows or high-magnification insets to demonstrate the expression by these different cells more clearly. Also, more specific details are required in the figure legend.*

The images provided are immunostains for KLF11. The WT-UUO section shows strong nuclear staining for KLF11 (dark brown stain). There is a glomerulus in the micrograph, which we have now indicated.

In the ureteric obstruction model, ligation of the ureter produces pressure induced dilation of tubules, which lead to tubular epithelial cell injury, interstitial fibrosis, tubular atrophy, and interstitial inflammation. Other compartments of the kidney (glomeruli, vasculature, etc.) are secondarily affected (see response to point 3, below).

*2. (Table 2) Was the expression of anti-inflammatory or regulatory genes (e.g., FoxP3, IL-10) profiled as well? Did the expression of these genes vary between groups?*

We did not find significant differences in anti-inflammatory or regulatory genes between WT and KO UUO groups.

*3. This reviewer suggests expanding the Discussion section to include details about how the authors believe KLF11 expression by various cellular subsets (podocytes, tubular epithelial cells, and monocytes) could reduce renal injury following UUO.*

We have expanded the discussion and added a reference to support our hypothesis that injury to KLF11 deficient tubular epithelial cells exacerbates pro-inflammatory and pro-fibrotic signaling pathways triggered by ureteric obstruction, a finding supported by our RNA expression data obtained from renal cortex.

In the ureteric obstruction model, the primary/initial target of injury is the tubular epithelial cell (see response to #1, above). In WT-UUO mice, we observed a strong induction of KLF11 staining, primarily within tubular epithelial cells. Based on this observation, we believe that injury to the tubular epithelial cell initiates a cascade of events leading to interstitial inflammation, interstitial fibrosis, and tubular atrophy. We propose that KLF11 deficiency exacerbates tubular injury through upregulation of pro-inflammatory and pro-fibrotic signaling pathways, as outlined in the results section.

Support for the notion that tubular epithelial cells can orchestrate pro-inflammatory and pro-fibrotic signaling pathways was obtained in our previous work using a murine model of renovascular hypertension initiated by placement of a cuff on the right renal artery, restricting blood flow by 75-80% (reference 26, added to address this concern). In this study, we subjected Ccl2-RFP reporter mice to renal artery stenosis surgery. We found a strong induction of Ccl2 (a potent chemoattractant for mononuclear cells) within tubular epithelial cells within 24 hours of renal artery stenosis surgery, a time point at which there was no significant tubular atrophy, interstitial fibrosis, or interstitial inflammation (see reference 26, added to this revision). Based on these considerations, we believe that the tubular epithelial cells—which are the target of UUO and show a striking increase in immunostaining for KLF11 following UUO—are responsible for the recruitment of inflammatory cells and initiate pro-fibrotic signaling pathways.

Minor Issues:
*1. (line 3) Suggest changing title from "ureteric" to "ureteral"*

Thank you for the suggestion. We have made the recommended change in line 3.

*2. (lines 293-295) Suggest changing interpretation of the immunohistochemistry images from "expression" to "infiltration" of macrophages.*

We have made the suggested change in line 292.

*3. (lines 306-307) Suggest defining CD163 as a marker. Is this meant to detect pro-inflammatory M1 macrophages?*

CD163 was used to detect pro-inflammatory M1 macrophages. This has been clarified in line 307-308.

*4. Suggest revision for English in the manuscript (especially the revised sections), as there are some errors in grammar and syntax.*

We have corrected errors in grammar and syntax.
